# Opportunities and Challenges for Antibodies against Intracellular Antigens

**DOI:** 10.7150/thno.35486

**Published:** 2019-10-15

**Authors:** Xiaofeng Yang, Shenxia Xie, Xiaomei Yang, Juan C. Cueva, Xiaoqiong Hou, Zhuoran Tang, Hua Yao, Fengzhen Mo, Shihua Yin, Aiqun Liu, Xiaoling Lu

**Affiliations:** 1 Nanobody Research Center, Guangxi Medical University, Nanning, Guangxi, 530021, China; 2 School of Preclinical Medicine, Guangxi Medical University, Nanning, Guangxi, 530021, China; 3 School of Stomatology, Guangxi Medical University, Nanning, Guangxi, 530021, China

**Keywords:** peptide, TCR-like antibody, MHC-peptide complex, CAR-T cell therapy

## Abstract

Therapeutic antibodies are one most significant advances in immunotherapy, the development of antibodies against disease-associated MHC-peptide complexes led to the introduction of TCR-like antibodies. TCR-like antibodies combine the recognition of intracellular proteins with the therapeutic potency and versatility of monoclonal antibodies (mAb), offering an unparalleled opportunity to expand the repertoire of therapeutic antibodies available to treat diseases like cancer. This review details the current state of TCR-like antibodies and describes their production, mechanisms as well as their applications. In addition, it presents an insight on the challenges that they must overcome in order to become commercially and clinically validated.

## Introduction

Cancer has caused tens of millions of deaths globally, making it the second leading cause of human deaths 1-3. In the ongoing war against cancer, immuno-oncology (I-O), a product of the many breakthroughs and discoveries in immunology and cancer therapy, has raised our hopes of improving cancer survival 4. In addition, I-O and its advancements were named the 2013's scientific breakthrough of the year by “science” 5. During the last few years, I-O based monoclonal antibody therapies have progressed at a rapid pace, the Food and Drug Administration (FDA) has approved over 50 monoclonal antibodies targeting PD-1, CTLA-4, CD30, CD20 and so on, which gave rise to now commercially and clinically available drugs such as Rituximab and Trastuzumab 6-8. The biological and pharmacological properties of monoclonal antibodies make them attractive to the researchers 9.

Regardless of the approach, the target antigens are mostly extracellular proteins 10, 11. This is partly due to the antibodies' high molecular weight that prevents them from crossing the cellular membrane and thus target intracellular antigens. Given that majority of the tumor-associated antigen (TAA) proteins produced by a cancer cell are produced intracellularly, the number of externally expressed tumor antigens is limited. What' s more, majority of the proteins identified as specific tumor markers are intracellularly localized such as the case of WT1, which is regarded as the most promising among the 75 representative target antigens 12, 13. Altogether, intracellular proteins may provide an untapped reservoir of potential therapeutic targets.

Many efforts have been made to target intracellular antigens; these strategies can be divided into two broad approaches. The first approach is to target the antigens that are normally intracellular but become externalized in exceptional situations, as in the case of heat-shock protein 70, heat-shock protein 90, phosphatase of regenerating liver 3 (PRL-3) and gp75 14-17. Zeng and colleagues focused on PRL-3, a cancer-related phosphatase which is undetectable in most normal human tissues but over-expressed in 85% of gastric cancers, and developed a humanized anti-PRL-3 antibody. The second approach is to engineer the antibodies or specific fragments to penetrate the cells or to express antibodies using a gene therapy approach. Some of these approaches include the usage of viral vectors, liposomes, nanoparticles and the fusion of peptides and antibodies 18-22.

Furthermore, intracellular proteins are degraded by the proteasomes to form short peptides of specific lengths which are normally 8-10 amino acids long. These peptides are then presented on the cell surface of the cancer cells in the context of major histocompatibility complex class I (MHC-I) molecules, forming various MHC-peptide antigens that can be recognized by T cells 23-25. Since the MAGE-1 gene was reported to encode a human tumor antigen recognized by T cells, molecular identification and characterization of novel tumor-associated antigens (TAAs) has expanded rapidly 26-31. To date, there are hundreds of identified MHC-peptide antigens, which can be used for the development of diagnostic methods and targeting therapy for cancer 32-34.

**Figure 1:**
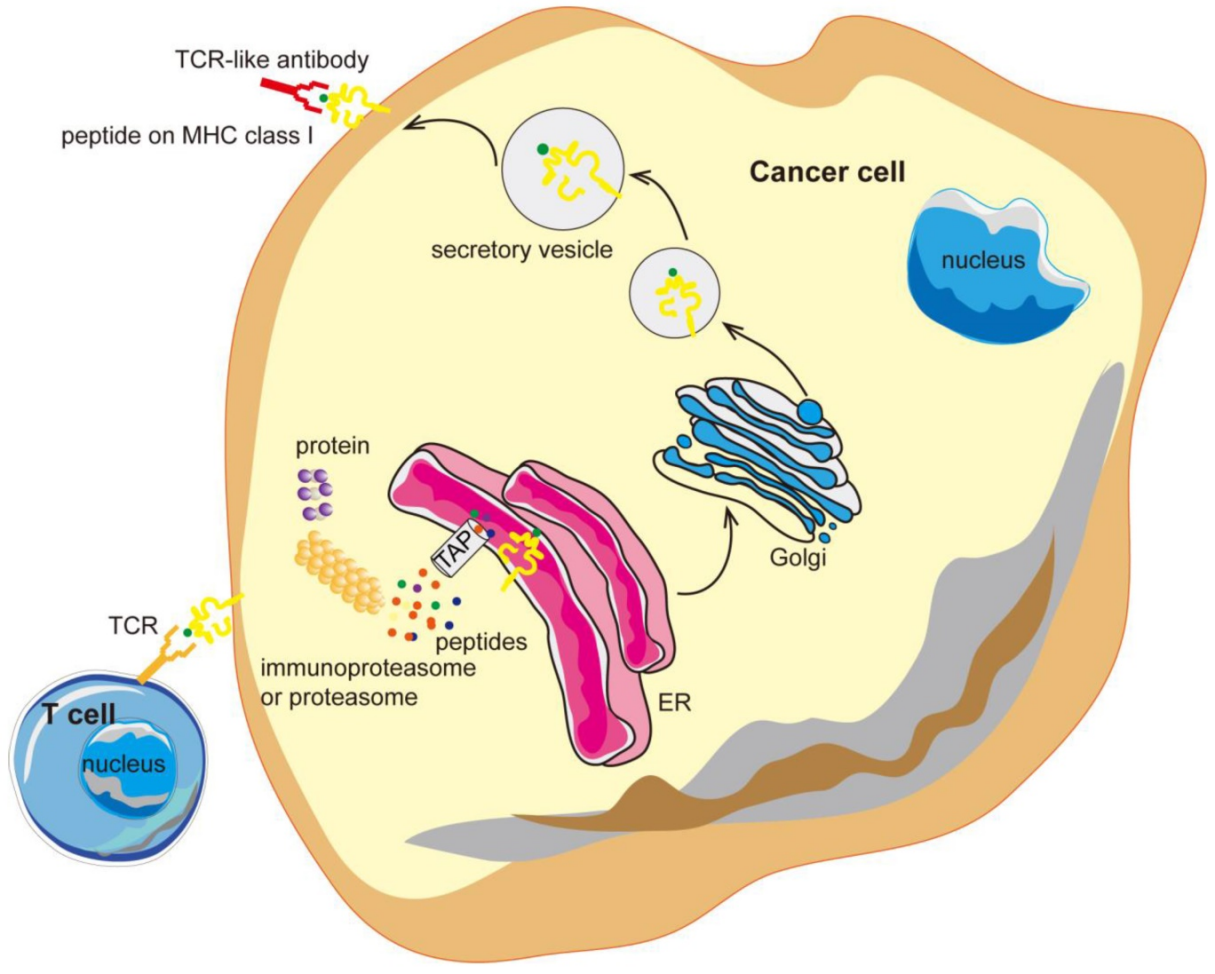
** TCR-like antibodies binding to specific MHC-peptide complexes on a cancer cell.** Intracellular proteins can be degraded by the proteasome and processed into peptides that are then presented on the cell surface in the context of MHC class I molecules. TCR-like antibodies can specifically target cancer cells exhibiting specific MHC-peptide complexes on their surface. MHC, major histocompatibility complex; ER, endoplasmic reticulum.

Antibodies targeting the MHC-peptide complexes are known as the T cell receptor mimic (TCRm) monoclonal antibodies (mAbs) or T-cell receptor (TCR) -like antibodies. TCR-like antibodies can combine the recognition of intracellular proteins (analogous to that of TCRs but with higher affinity) and the therapeutic potency as well as the versatility of mAbs 35, 36. TCR-like antibodies are redefining the selection of suitable targets in cancer therapy and may open the door to a new realm of antibody therapy, with promising clinical benefits (Figure 1).

### Production of TCR-like antibodies

During the last decades there has been significant progress in the development of TCR-like antibodies, and several research groups have been able to generate TCR-like antibodies directed to a growing repertoire of cancer. The traditional production manner of TCR-like antibodies is hybridoma technology. The first and most important step using conventional hybridoma is to obtain purified recombinant MHC-peptide complexes that are recognized by a T cell 37, 38. These complexes are prepared by the means of bacterial expression (usually *Escherichia coli.*) to generate inclusion bodies comprising the extracellular domain of the heavy chain of human leukocyte antigen (HLA) and β2-microglobulin. Thence, the HLA heavy chain and 2-microglobulin inclusion bodies are refolded in the presence of the desired HLA-restricted peptides *in vitro* 39-41. The refolding must be processed in the right conformation, as demonstrated by structural and functional studies, then these complexes can be used for downstream applications 42.

At first, researchers employing traditional hybridoma technology used antigen-presenting cells (APCs) presenting strong immunogenic peptides in their MHC complex as immunogens but not the purified recombinant MHC-peptide complexes 43, 44. The peptide-specific, MHC-restricted antibodies using the above-mentioned technique are quite rare even under optimal conditions; given that one to three out of 1000 growth positive clones could produce antibodies of the requested specificity 45-50. Although the attempts to improve this technique have failed several times, numerous research groups have used recombinant MHC-peptide complexes for the isolation of TCR-like antibodies and have been successful in using conventional hybridoma technology 51-55.

**Figure 2:**
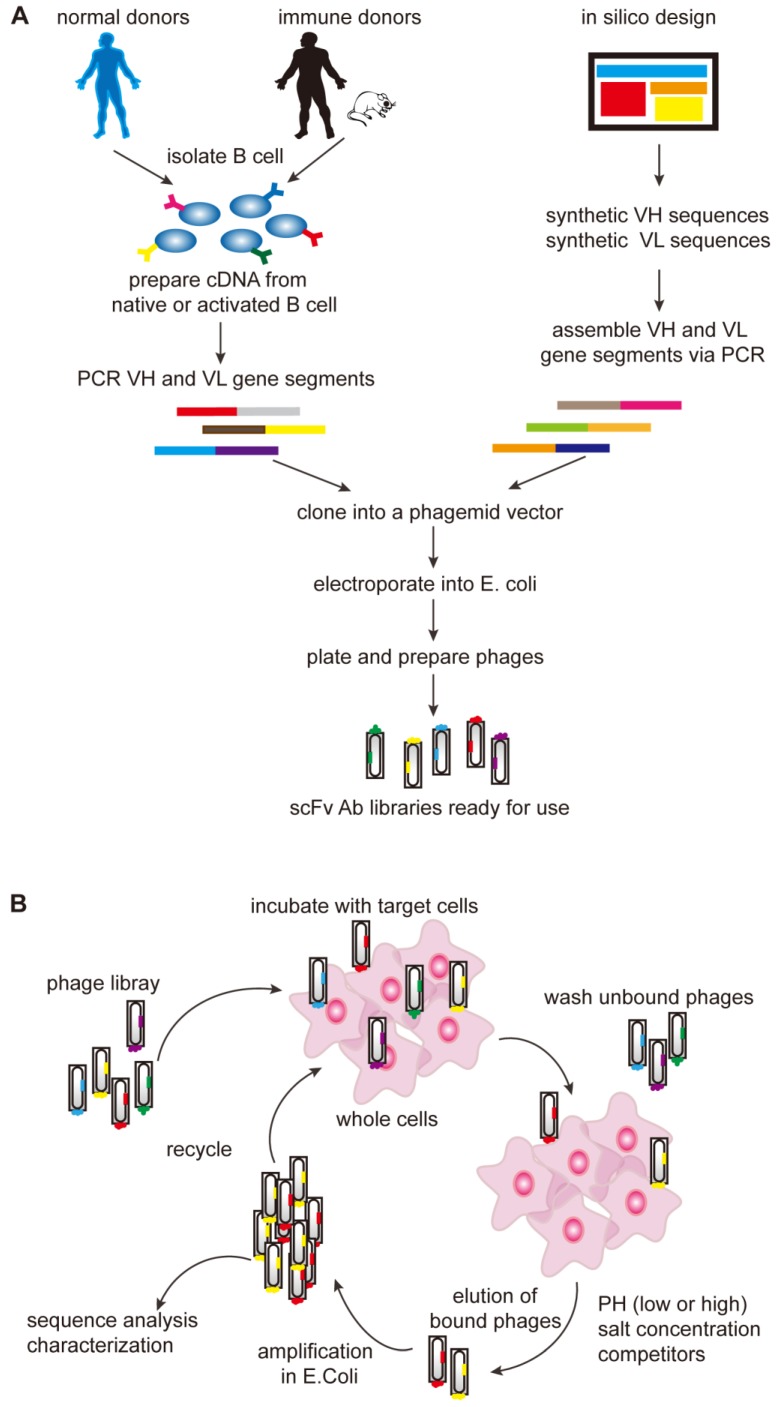
** Generation of antibody libraries and selection of the TCR-like antibodies. (A)** Diverse repertoires can be obtained from the rearranged V-gene segments which are derived either from naïve or activated B cells subsequent to immunization or infection or human V-gene segments rearranged *in vitro* (synthetic repertoires). The assembled scFv/Fab repertoires are then cloned into a phagemid vector in order to be expressed on the surface of the phage as single-chain scFv or Fab antibody libraries and then the phage library is incubated with the desired target cells. **(B)** After incubation, there are two types of the phages. The unbound phages are removed through washing and the bound phages are eluted and propagated in E. coli. The bound phages are then used for further rounds of selection to get the specific binders. Fab, fragment antigen binding; scFv, single-chain variable fragment.

In the mid-1990s, it was shown that phage display technology could also be used to isolate antibodies (Figure 2). The pioneering work by Andersen and colleagues demonstrated that phage-display could be used as a tool to isolate antibodies with unique specificity, furthermore, subsequent works consecutively proved phage-display technology to be a promising way to isolate antibodies 56. The first step of phage-display is to generate antibody libraries exposed as fusion proteins on the surface of phage particles. These libraries are called the naïve libraries, and each phage particle in the naïve libraries displays a unique antibody. Phage particles carrying specific antibodies are purified by repeated rounds of selection, and then TCR-like antibodies (scFv/Fab) are isolated from large naïve human phage-display libraries 57-60. The phage-based approach can be consistently applied to isolate recombinant antibodies with the requested specificity, providing new means for TCR-like antibodies production.

The affinity of TCR-like antibodies isolated from a naïve phage-display library is not always sufficient for therapeutic purposes. Similar to hybridoma technology, many efforts have been made to improve the affinity of the TCR-like antibodies, second-generation libraries generated by different affinity-maturation strategies are used for the isolation of the TCR-like antibodies. Chames and colleagues isolated an 18-fold affinity TCR-like Fab (the VH-VL hybrid clone Hyb3) directed to the cancer T-cell peptide HLA-A1-MAGE-A1, using Fab G8 as the platform for the construction of two randomized libraries: L chain shuffling library and H chain complementarity determining region 3 mutated library 61. Renner and colleagues have achieved the 20-fold affinity improvement of a new TCR-like Fab to the HLA-A-0201-NY-ESO-1 peptide using a second-generation Fab library. This Fab library is based on Fab 2M4E5 in which they randomized residues at positions that could optimize peptide interaction to improve their affinity, without changing the key residues responsible for the binding of the complex antigen 62. It was also reported that using transgenic mice expressing the desired human MHC allele on a murine MHC knocked out background would increase the probability of isolating a rare TCR-like antibody 63.

Most of the TCR-like antibodies published works have used phage display for antibody production 64-68. The major advantage of the phage display approach is the high selection power of the desired antigens due that the process is being achieved within a relatively short time, conversely the generation of TCR-like antibodies using hybridoma technology is less efficient and relatively more time consuming 69. TCR-like antibodies isolated using hybridoma technology were reported to have higher binding affinity compared to the moderate average affinity of TCR-like antibodies isolated from the naïve phage display libraries 70, 71. Therefore, using hybridoma technology could have a tendency for isolating antibodies with high-affinity binding to the MHC-peptide complexes. The antibodies produced by hybridoma technology are bivalent IgG isotype antibodies while antibodies produced by phage display are either scFv or Fab fragments. IgG antibodies are more stable and have a superior affinity due that antibodies undergo multiple antigen challenges and affinity maturation *in vivo*. Antibodies in the monovalent form have reduced avidity (functional affinity) and increased turnover rates, which are undesirable when targeting peptides on tumor-associated MHC-peptide complexes. Although monovalent forms are useful reagents for a variety of TCR-like applications, their reduced binding strength is a great limitation. To overcome this limitation, it is reported that Fab or scFv-tetramers or transformer of IgG isotype can improve the binding avidity 72-75.

Production techniques for TCR-like antibodies are not shared, and most phage display libraries are proprietary. Tao Dao and colleagues have discovered a fully human “T cell receptor-like” monoclonal antibody (mAb) ESK1, specific for the WT1 RMF peptide/HLA-A0201 complex. The antibody ESK1, developed in collaboration with Memorial Sloan Kettering Cancer Center (MSKCC) and Eureka Therapeutics, is now patented. Eureka Therapeutics, a pioneer in the development of TCR-like antibodies; currently works with different companies to develop TCR-like antibodies against different intracellular proteins. Bringing the developed TCR-like antibodies into clinical trials as soon as possible is one of the most notable challenges faced by all researchers in this area.

### TCR-like antibodies to date

Over the past 20 years, there has been an increase in the production of TCR-like antibodies. Initially most of the target peptides of these antibodies were derived from viruses; however, with the introductions of TCR-like antibodies, new key targets for the treatment of cancer have been discovered 76-83. Having two different production strategies, TCR-like antibodies can be bivalent IgG isotype antibodies produced through hybridoma technology or the scFv or Fab fragments produced through phage display. Regarding IgG isotype antibodies, most of them were produced by four laboratories. Weidanz's research laboratory engineered RL4B/3.2G1 targeting HLA-A2-restricted GVL peptide derived from the parent protein hCGβ, 1B8 targeting HLA-A2-restricted KIF peptide derived from parent protein Her2/neu in 2006, 1B10/3F9 targeting HLA-A2-restricted GVL peptide/TMT peptide derived from parent protein hCGβ in 2008, RL6A targeting HLA-A2-restricted YLL peptide derived from parent protein p68 in 2010, and RL21A targeting HLA-A2-restricted MIF19-27 peptide in 2011 84-88. Banham's research laboratory engineered T1-29D/T1-84C/T1-116C targeting HLA-A2-restricted RMP peptide derived from parent protein p53, and T2-108A/T2-2A/T2-116A targeting HLA-A2-restricted GLA peptide derived from parent protein p53 in 2017 89, 90. Molldrem's research laboratory engineered a TCR-like antibody 8F4 targeting HLA-A2-restricted VLQ peptide derived from parent protein Proteinase3 in 2011 91, 92. Scheinberg's research laboratory engineered the antibody ESK1 targeting HLA-A2-restricted RMF peptide derived from parent protein WT1 in 2013, and its Fc enhanced form ESKM in 2014 93, 94.

The scFv or Fab fragments produced through phage display are not complete IgG isotype antibodies and they cannot recruit components of the immune system for cytotoxic effects through antibody-dependent cell-mediated cytotoxicity (ADCC) and complement dependent cytotoxicity (CDC). Literatures regarding TCR-like antibodies like scFv or Fab fragments published to date can be divided in three groups.

The first group focuses on the selection and characterization of the Fab/scFv fragments, researchers used naked TCR-like antibodies to target cells. Patrick Chames and colleagues engineered a fully human Fab fragment Fab-G8 directed against the HLA-A1-MAGE-A1 complex by selection from a large naïve phage-antibody library in 2000, posteriorly they enhanced TCR-like antibody Fab-Hyb3 by selection from a second-generation library in 2002 61, 95. Galit Denkberg and collaborators engineered Fab fragments like 1A7 from a large nonimmune repertoire of phage Fab Abs in 2002 96. Avital Lev and collaborators finished the isolation of some human antibodies with antigen-specificity, MHC-restricted specificity of T cells binding with HLA-A2 complexes which display the specific hTERT-derived peptide 97. Christoph Renner's laboratory described the selection and characterization of Fab fragments recognizing the NY-ESO-1157-165 peptide in the HLA-A*0201 context 98. Renner's laboratory also selected Fab antibodies binding to the HLA-A2-restricted EAA or ELA peptide derived from the parent protein Melan-A 99.

Similarly, articles from the second group describe the selection and characterization of the Fab/scFv fragments, however in this case researchers modified the structure of the TCR-like antibodies. Galit Denkberg and colleagues reported for the first time the fusion of the TCR-like antibody gene to a truncated form of Pseudomonas exotoxin A to form a recombinant immunotoxin 63, 100. Cyril J. Cohen and colleagues reported the production of fluorescent tetramerized Fabs to directly visualize and quantitate the specific HLA-A2/MUC1-D6 peptide on the surface of tumor cells 101. David A. Scheinberg's laboratory selected a ScFv fragment that is specific for the WT1 RMF peptide/HLA-A*0201 complex found on many human cancers, they also engineered the scFv fragment to a full length human monoclonal antibody to target cancer cells 93. The three before mentioned reports are the main representatives of this type of literatures and there are also some papers reporting similar work 102-104.

The third group describes the selection and characterization of Fab/scFv fragments, in this articles researchers describe the development of TCR-like antibodies into a CAR based approach on the TCR-like antibody of a high-affinity antibody that recognizes the complex antigen composed of the MHC molecule and a peptide derived from the antigen protein. David A. Scheinberg's laboratory selected a scFv fragment that is specific for the WT1 RMF peptide/HLA-A*0201 complex and then created a TCRm CAR against WT1 utilizing the previously described scFv which demonstrated effective *in vitro*/vivo efficacy 105. WT1 is over-expressed in numerous hematological malignancies like acute myeloid leukemia (AML), as well as in many solid malignancies such as ovarian cancer, thus the created WT1 TCRm CAR T-cell approach allows for the application of a single CAR to a wider array of malignancies 106-108. Hong Liu and colleagues developed ET1402L1, a fully human antibody that selectively binds to AFP158-166 (AFP158) peptide presented by HLA-A*0201, then engineered this antibody into a second generation CAR, demonstrating that CAR-T cell immunotherapy targeting intracellular/secreted solid tumor antigens like AFP can elicit potent anti-tumor responses 109-112. Similar works targeting different peptides presented by HLA-A molecules have been reported 113-118.

### Mechanisms of the TCR-like antibody function

Similar to monoclonal antibodies that target specific tumor antigens, naked TCR-like antibodies can also be used to mediate ADCC and CDC, which are also called the Fc-dependent mechanisms of mAb. RL4B and other naked TCR-like antibodies have been shown to induce CDC mechanisms *in vitro*, which are induced by the binding of the complement proteins to the Fc region of therapeutic TCR-like antibodies, however most of the naked TCR-like antibodies have been proved to induce ADCC mechanisms *in vitro* 119-122. The exact mechanism of ADCC varies depending on the type of immune effector cells that are activated. Activated NK cells secrete perforin and granzyme B, which are taken up by the target cells and result in their lysis, while monocytes and macrophages are capable of secreting cytotoxic factors like TNF and reactive oxygen intermediates 123-125.

Naked TCR-like antibodies like RL4B can also to deliver a direct apoptotic signal to cancer cells in a mechanism involving JNK activation and the caspase-dependent pathway, directly killing the target cells 126. Similar to the Fc-independent killing mechanism, immunoconjugates such as immunotoxins and immuno-drugs are able to directly kill the target cells. The targeting moiety of the immunoconjugates can be a Fab or a scFv fragment composed of the variable domains VH and VL which are covalently linked through a peptide linker 127-132. Immunotoxins or immuno-drugs' mechanisms of action allows them to directly kill cancer cells, conferring them a clinical benefit in the treatment of patients who may not respond to agents that require a fully functioning immune system.

**Figure 3:**
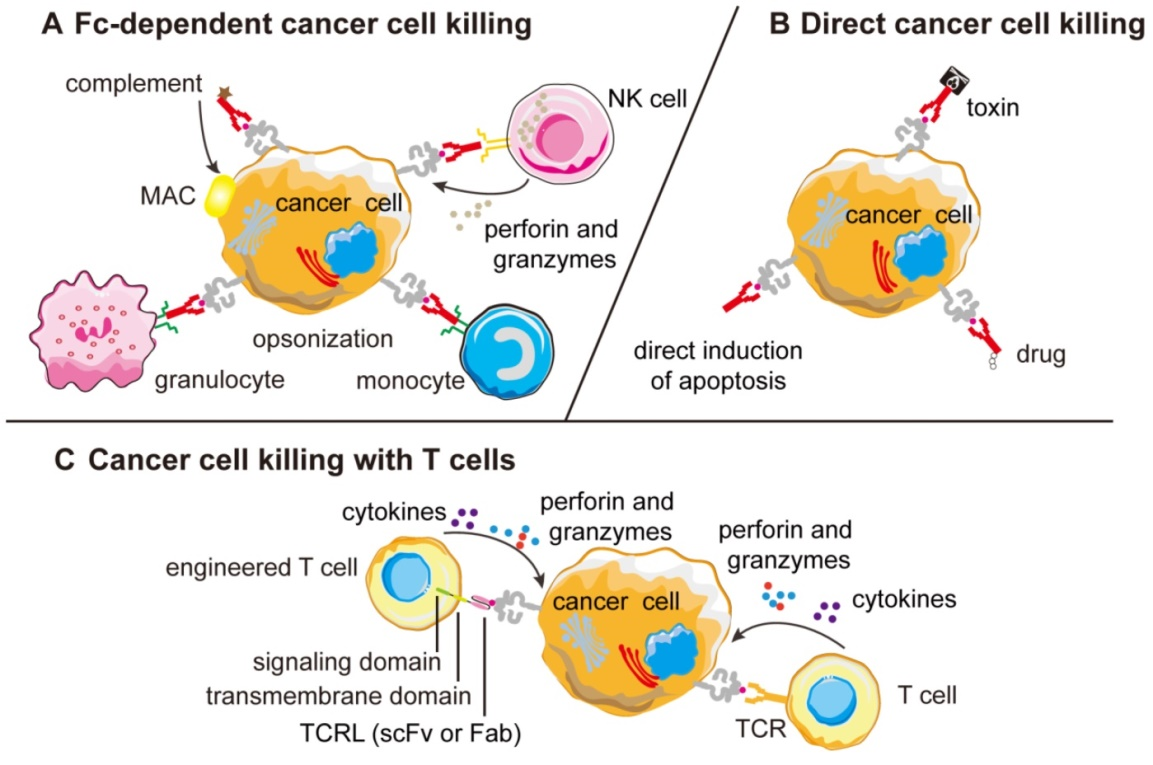
** Mechanism of action of TCR-like antibodies against cancer cells. (A)** Most naked TCR-like antibodies induce CDC or ADCC mechanisms that are Fc-dependent and the ADCC mechanism can be different among different effector cells. **(B)** Naked TCR-like antibodies can also induce apoptosis mechanism. When fused to toxins or drugs, the fusion protein can kill the tumor cells directly. **(C)** T-cells engineered to display TCR-like antibodies as receptors can re-direct cytotoxic T cells against cancer cells forming lytic immunological synapse. CDC, complement-dependent cytotoxicity; MAC, membrane attack complex; ADCC, antibody-dependent cell mediated cytotoxicity; Fab, fragment antigen binding; scFv, single-chain variable fragment; MHC, major histocompatibility complex; TCRL, T-cell receptor-like.

In adoptive T-cell transfer therapeutic approaches like CAR-T cell therapy, autologous T cells are isolated, expanded and engineered *in vitro* and re-infused to patients 133-135. TCR-like antibodies are responsible for recognition, while cytotoxic T-cell signaling moiety FcεRIγ chain is responsible for the initiation of tumor-specific killing activities and cytokines release 136, 137. The engineered T cells were found to specifically bind MHC-peptide complexes on target cells, leading to the production of cytokines and induction of cytolysis (Figure 3).

### The applications of TCR-like antibodies

TCR-like antibodies can be used to directly visualize the presence of MHC-peptide complexes by standard methods such as flow cytometry 138-140. Since TCR-like antibodies can provide novel data regarding antigen presentation in various cells, TCR-like antibodies can be used to analyze immunotherapy-based approaches by determining the alterations in MHC-peptide complexes expression on cells before, during and after the therapies, this could also provide new powerful means to study the structure-function relationships in the MHC-peptide context 141-149. Since the density of a particular MHC-peptide complex on tumor cells is expected to be low compared to peptide-pulsed or transfected APCs, TCR-like antibodies were engineered to make tetramers, with directly tagged fluorescent probes 150.

**Figure 4:**
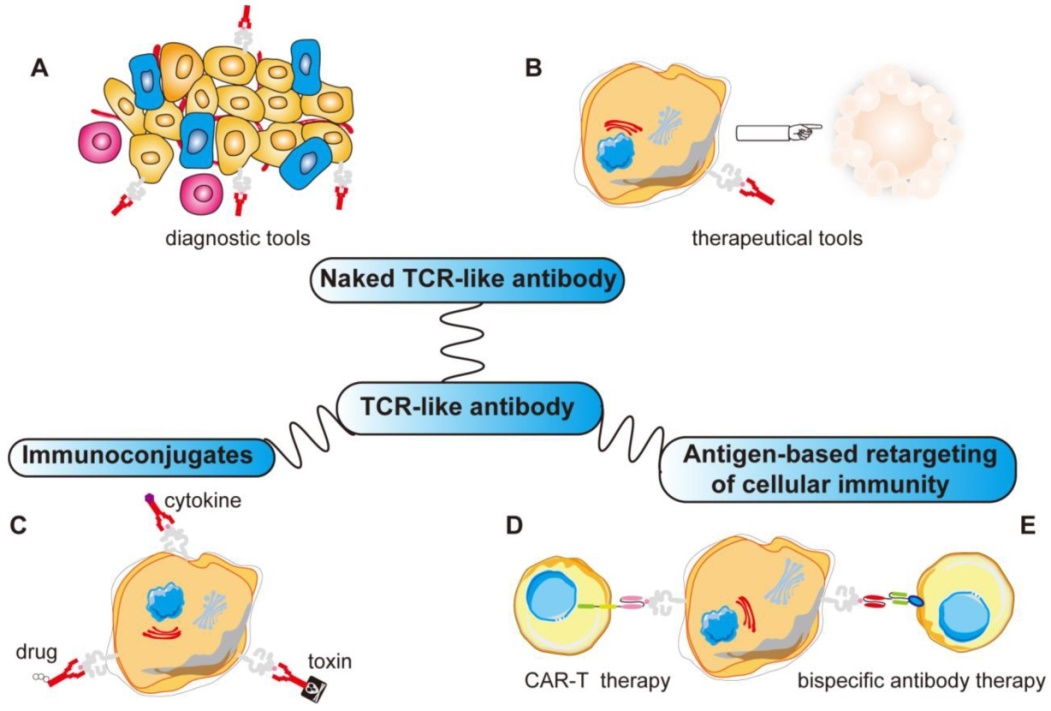
** Various applications of TCR-like antibodies.** Fab/scFv fragments with MHC restricted specificity obtained by phage display can be used in different ways: **(A, B)** Directly to target the specific MHC-peptide complexes as diagnostic tools or therapeutic tools. **(C)** Fused to a drug/toxin/cytokine to form immunoconjugates. **(D)** Fused as a signaling moiety to genetically retargeting T cells toward cancer cells. **(E)** Reformated as bispecific antibody binding simultaneously a MHC-peptide complex and a receptor expressed by effector cells (CD3 on T cells). ADCC, antibody-dependent cellular cytotoxicity; CDC, complement-dependent cytotoxicity; Fab, fragment antigen binding; scFv, single-chain variable fragment; MHC, major histocompatibility complex.

More importantly, TCR-like antibodies present new opportunities for use as targeting moieties for various antibody-based immunotherapeutic approaches because of their exquisite specificity towards a very precise and unique human tumor antigen. This includes using such antibodies to construct recombinant immunotoxins/drugs, fusion with cytokine molecules, bispecific antibody therapy and for CAR-T therapy 151-160. With more applications yet to be explored, TCR-like antibodies promise to be as useful as monoclonal antibodies (Figure 4).

### Choosing the ideal target

Tumor associated antigens can be classified into three categories: cancer testis antigens and oncofetal antigens, differentiation antigens and over-expressed antigens. Cancer testis antigens and oncofetal antigens like MAGE, WT1 as well as alpha-fetoprotein are expressed in a wild range of different cancer cells, but limited in normal tissues except for embryonic cells or germ cells 161-164. Differentiation antigens are restrictively expressed in limited cell lineages, such as gp100, Tyrosinase, and MART-1 165-168. Over-expressed antigens are normal proteins, but over-expressed or amplified in cancer cells, such as PR1, Her2/neu, PSA, EGFR 169-176. They are all potential cancer targets, and they have their own pros and cons. Targeting the differentiation antigens like KRAS G12V/D is the most conceptually interesting approach as it can kill specific cancer cells without injuring normal cells, nevertheless they are also difficult to rationalize for therapeutic drug development 177. Targeting the cancer testis antigens and over-expressed antigens like NY-ESO-1 and Her2/neu could also be a more general strategy, although the expression on the normal healthy tissues must be considered 178, 179.

The process of proteins expression and the presentation of short peptides on MHC are well described. However, the specific rules that govern which protein peptides are ultimately presented on the cell surface are poorly understood and therefore not fully predictable. Further identification of the presented peptides is an empirical process 180, 181. In cancer, the discovery of these peptides mostly emerged from the observation that cancer cells express antigens which can be recognized by cytotoxic T-lymphocytes (CTLs) derived from patients 182-189. Some peptides may be generated but have low affinity to MHC, while other peptides may have high affinity to MHC molecule, but never reach the cell surface due to improper processing 190. Hence, many potentially interesting targets are not available as MHC-presented cell-surface peptides.

Databases such as the Cancer Genome Atlas can be used to identify mutated protein sequences that can be used as potential targets, but current methods to identify peptides presented by cancer cells on surface MHC may lack the required sensitivity, and thus are able to identify only a limited number of possible antigens 191, 192. Therefore, some peptides that are likely to be TCR-like antibody targets, may not be detected. cDNA expression cloning is used as the original technique for isolating tumor antigens recognized by CD8+ T cells, but with this method is difficult to determine MHC restriction for unique antigens. Posteriorly, SEREX (serological analysis of recombinant cDNA expression libraries), cDNA expression cloning using serum IgG Ab from cancer patients, was developed. But RNA expression levels do not correlate with that of protein expression, in other words, RNA expression levels do not concord with peptide presentation levels. Therefore, mass spectrometry is the direct way to identify peptides presented by cancer cells on surface MHC, and it has been the consensus in the field 193.

Although both TCR-like antibodies and traditional antibodies can bind to antigens, the binding sites and methods are different. Traditional mAbs bind to conformational antigens, whereas TCR-like antibodies recognize complex antigens composed of MHC molecules with embedded short peptides. For a given haplotype, the MHC component is invariant, and the embedded peptides can come from the millions of sequences encoded in the exome. Most TCR-like antibodies have been shown to bind only a few residues of their target linear peptide 194-196. This suggests that TCR-like antibodies may theoretically have many off-target peptides that share the same residues at major contacts, but differ on other positions. For instance, the therapeutic TCR-like antibody ESK1 only binds to complex antigens composed of HLA-A*0201 amino acids in addition to 3-5 N-terminal residues of the WT1-derived peptide 9-mer. Exchange of the C-terminal amino acid of the target peptide still allows binding of ESK1 TCR-like antibody. On the contrary, a TCR-like antibody targeting the cancer-testis antigen PRAME was shown to bind the C-terminus of the full-length sequence as well as TCR-like antibody targeting the tumor-associated antigen PR1 that was shown to depend heavily on one residue of the PR1-peptide 197, 198.

### Enhancing peptide density

The peptide density of TCR-like antibody targets has been reported to hold only 100-1,000 sites per cell, which is significantly lower than some reported peptide densities of conventional monoclonal antibody whose cell surface targets are 20,000-500,000 sites per cell. The density of TCR-like antibody cell surface sites is positively correlated with the killing effect of TCR-like antibodies on target cells, so a key factor that needs to be considered when choosing a target antigen for TCR-like antibody therapy include the peptide density on the cell surface. The levels of protein expression and presentation, HLA levels, protein half-life, levels of MHC-peptide complex presentation all dictate TCR-like target peptide density.

A sufficient amount of protein must be translated to facilitate peptide processing. For example, the hypomethylating agent Decitabine can significantly increase the expression of NY-ESO1 in patient's tumor biopsies 199. Another methyltransferase inhibitor, 5-azacytidine, can also induce cancer-testis antigen-specific CTLs in patients, minimally affecting immune effector populations and function 200. Protein stability also plays an important role, it is reported that defective ribosomal products (DRiPs) that can be degraded quickly constitute a large percentage of peptides presented on MHC, and they may accumulate due to errors in the process of transcription, translation, or protein folding. Short-lived proteins appear to be more likely than proteins with longer half-lives 201.

After the expression of the proteins, they are cleaved into random-sized peptides by proteasomes in the cytosol. Proteasomes are the major complex that degrades proteins into peptides, constitutive proteasome (CP) or the immuno-proteasome (IP) are the two major forms of proteasomes 202, 203. Hence, modulation of proteasome expression by treatment with cytokines or proteasome inhibitors could enhance or eliminate the presentation of specific peptides 204. To name some examples, Lactacystin is an organic compound naturally synthesized by bacteria of the genus Streptomyces, the influenza M1 58-66 peptide is more efficiently produced in the presence of Lactacystin, and a significant number of the cancer antigen MAGE-3 271-279 is presented by melanoma cells after the inhibition of Lactacystin 190, 205.

Posterior to degradation, these cytosolic peptides are then pumped into the endoplasmic reticulum through TAP, where they are trimmed by aminopeptidases, loaded onto MHC molecules, and transported to the cell surface. Peptide MHC loading is governed by the binding affinity of the MHC protein to the peptide. HLA molecules are not internalized readily, and it has been reported that up to 90% MHC class I down-regulation has been noted in several cancers 206. Given all these obstacles, down regulation of the MHC class I expression prevents a properly processed peptide from reaching the cell surface. Using some agents to upregulate the MHC class I expression is necessary. MEK inhibitor PD98059 has been reported to increase MHC expression in esophageal and gastric cancers through the inhibition of the MAPK pathway 207. Similarly, the EGFR inhibitor Erlotinib could induce increased MHC class I levels on patients treated with it 208. In addition to small molecule modulation of the inhibitor, targeting associated protein molecules can also increase expression of MHC molecules. For example, β2 microglobulin (β2M) is often downregulated in cancers, using the modulators of β2M may have the possibility to increase target peptide presentation 209.

### Future for the TCR-like antibodies

Based on the fact that T-cell receptor mimicking antibodies have not yet entered the clinic, several key factors have the potential to improve the development of TCR-like antibodies so that TCR-like antibodies can have the prospect of undertaking clinical studies and ultimately establish themselves as a type of cancer therapy.

Antibodies with the MHC-restricted specificity of T-cells are rare and lots of high-affinity, peptide-specific TCR-like antibodies have proven to be difficult to produce by either hybridoma approaches or phage display because B cells are not educated to be self-MHC restricted 210-214. T cells are educated to recognize antigenic peptides presented in complex with MHC class I or II molecules through the alternating selection processes, while B-cells are not in this selection process. The creation of TCR-like antibodies is expensive and time consuming. In order to isolate fully high-affinity, peptide-specific human antibodies within a short period of time, the improvement of traditional production methods and even the creation of new production methods are necessary.

It is crucial to ensure that the TCR-like antibodies do not recognize the MHC-I alone, as this molecule is found on most nucleated cells, and it does not cross-react with other processed peptides, as TCR-like antibodies recognize only a few amino acid residues in the peptide, which means that other processed peptides possess the same amino acids at those positions 215, 216. Therefore, the TCR-like antibody must be specific for the specific peptide-MHC complex. A clinical trial of an affinity-enhanced TCR, which targeted a MAGE-A3 peptide, was reported to cause two patient deaths 217. It was discovered that the TCR also recognized a peptide on the unrelated protein titin that is expressed in cardiac tissue which was not observed in normal tissue screening and was not conserved in mice. The lack of suitable animal models to study whether TCR-like antibodies can target other cross-reactive peptides *in vivo* is a major problem disrupting its clinical applications.

One of the key limitations of TCR-like antibody therapy is the MHC restrictive nature of treatment, although it is vital to be able to recognize intracellular proteins. Most studies to date focused on the HLA-A*0201 haplotype, which is found in up to 40% of Caucasians and 10-20% of other ethnic groups around the world 218-220. There are other dominant HLA alleles such as HLA-A*2402. Although TCR-like antibodies are HLA-restricted, it has been proposed that antibodies to three HLA alleles for a particular target antigen would cover over 96% of the world's population, it is reported that TCR-like antibodies bind multiple HLA-A*02 variants and not only the HLA-A*0201 subtype, suggesting that certain TCR-like antibodies could target a larger population of patients with a variety of HLA subtypes 221, 222. Therefore, HLA-A restriction does not limit the treatment to a limited number of patients, and the use of HLA-restricted presentation peptides allows multiple antibodies to be designed for specific antigens for combination therapy that could achieve better results.

There are a lot of strategies to augment the therapeutic index of the TCR-like antibodies. In addition to increasing the expression of complex MHC-peptide antigens, the application of TCR-like antibodies to other therapeutic methods and the combination with other treatments are the future direction. TCR-like antibodies can serve as an ideal cancer targeting platform for the delivery of cytotoxic payloads specific to tumors such as potent drugs and toxins. TCR-like antibodies can also be engineered into bispecific T-cell engagers (BiTEs) and chimeric antigen receptors (CARs) for expression on cytotoxic T-cells 223-225. CARs are single-chain variable fragment (scFv)-based receptors used to redirect T cells to recognize and lyse cancer cells. CARs would be advantageous in that they do not directly compete with the native TCR, and would further provide supportive co-stimulation to the transduced T cells 226. In 2018, The FDA made history by approving the first gene therapy in the United States. Kymriah, a cell-based gene therapy, is approved in the United States for the treatment of patients up to 25 years of age with B-cell precursor ALL that is refractory or in second or later relapse. On October 18^th^ of the same year, the FDA approved the Kite Yescarta (axicabtagene ciloleucel, KTE-C10) cell gene therapy for the treatment of diffuse large B-cell lymphoma 227, 228. More importantly, Joseph A. Fraietta and colleagues reported that due to the proliferation of a single CAR-T cell, a patient with chronic lymphocytic leukemia (CLL) treated with CAR-T cells in 2013 was relieved and remains cancer-free for over 5 years, and that the CAR-T cells are still present in his immune system 229. Due to the great therapeutic potential of CAR-T therapy, the application of TCR-like antibodies to CAR-T will make TCR-like antibodies the ideal companion for this role.

**Figure 5:**
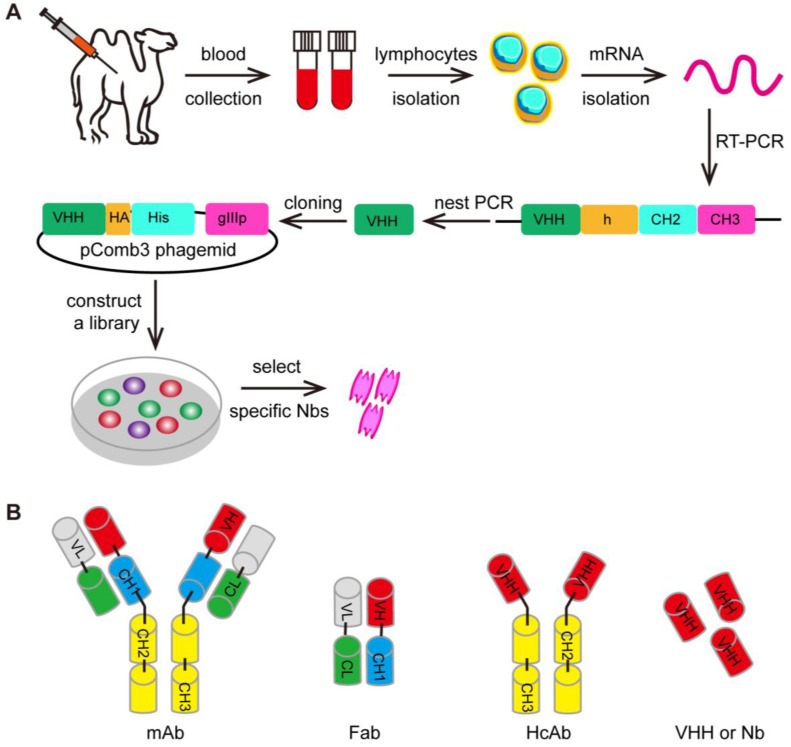
** The selection of the VHH. (A)** The first step of the selection using phage-display technology is to generate VHH libraries. After repeated rounds of selection, the specific VHHs are isolated from the large libraries. **(B)** The various antibody formats: mAb (monoclonal antibody), Fab (fragment antigen binding), HcAb (camel heavy-chain antibody), VHH or Nb (nanobody).

Furthermore, the variable (V) region domain can be used by its own to form a domain antibody called nanobody (Figure 5) 230. These nanobodies can be engineered from the heavy-chain antibody (HcAb) derived from camelids (camel or llama) or cartilaginous fish (carpet or nurse sharks), whose immune systems were found to have evolved high-affinity V-like domains that do not require intramolecular disulfide bonds for stability 231-234. The ability to specifically recognize unique epitopes with sub-nanomolar affinity have made nanobodies a useful class of biomolecules for medical research due to their various diagnostic and therapeutic applications. To name some recent examples, nanobodies have been employed as a cell re-targeting moiety in CAR-T cell therapy to target the extracellular antigens 235-237. It has also reported that TCR-like CARs containing GPA7, a single-domain antibody (sdAb) specific for gp100 209-217/HLA-A2 complex, could mediate the enhanced cytotoxicity of transgenic T cells against HLA-A2-matched melanoma *in vitro* and *in vivo* 117, 238. The variable (V) region domains represent the smallest format of the antibody that retains target specificity and it can replace traditional antibodies in a series of applications for cancer treatment.

## Conclusion

Expanding the targeting repertoire to the intracellular proteome represented by the MHC molecules, the generation of antibodies that can target intracellular antigens offer unparalleled opportunities not only for optimizing cancer treatment but also for the development of new anticancer strategies. TCR-like antibodies transform the fine cellular specificity of the T-cell recognition into an antibody-based immunotherapeutic approach and also fit in with the growing field of personalized medicine. The vast new arrays of potential targets presented by the MHC molecules suggest that TCR-like antibodies will find an important place in our armamentarium, picturing a promising next step for immunotherapy.
